# Can AI reveal the next generation of high-impact bone genomics targets?^☆^

**DOI:** 10.1016/j.bonr.2025.101839

**Published:** 2025-03-24

**Authors:** Casey S. Greene, Christopher R. Gignoux, Marc Subirana-Granés, Milton Pividori, Stephanie C. Hicks, Cheryl L. Ackert-Bicknell

**Affiliations:** a Department of Biomedical Informatics, University of Colorado School of Medicine, Aurora, CO, USA; b Colorado Center for Personalized Medicine, University of Colorado Anschutz Medical Campus, Aurora, CO, USA; c Department of Biostatistics, Johns Hopkins Bloomberg School of Public Health, Baltimore, MD, USA; d Department of Biomedical Engineering, Johns Hopkins University, Baltimore, MD, USA; e Center for Computational Biology, Johns Hopkins University, Baltimore, MD, USA; f Malone Center for Engineering in Healthcare, Johns Hopkins University, Baltimore, MD, USA; g Colorado Program for Musculoskeletal Research, Department of Orthopedics, University of Colorado School of Medicine, Aurora, CO, USA

**Keywords:** Machine learning, Artificial intelligence, Genetics, Systems biology, Target discovery, Knowledge graph

## Abstract

Genetic studies have revealed hundreds of loci associated with bone-related phenotypes, including bone mineral density (BMD) and fracture risk. However, translating discovered loci into effective new therapies remains challenging. We review success stories including PCSK9-related drugs in cardiovascular disease and evidence supporting the use of human genetics to guide drug discovery, while highlighting advances in artificial intelligence and machine learning with the potential to improve target discovery in skeletal biology. These strategies are poised to improve how we integrate diverse data types, from genetic and electronic health records data to single-cell profiles and knowledge graphs. Such emerging computational methods can position bone genomics for a future of more precise, effective treatments, ultimately improving the outcomes for patients with common and rare skeletal disorders.

## 1 Introduction

Human genetics has a track record of informing successful therapeutic development efforts (Nelson et al., 2015; King et al., 2019; Sabatine et al., 2017). For the last decade, it has been clear that drugs with support from human genetics are more likely to show efficacy than those without such support (Nelson et al., 2015). Updated estimates continue to support this strength (Minikel et al., 2024). The example of Proprotein convertase subtilisin/kexin type 9 (*PCSK9*) demonstrates how genetic evidence can guide therapeutic design. Variants in *PCSK9* were initially found to segregate with autosomal dominant hypercholesterolemia (Abifadel et al., 2003), and further investigation of the gene revealed nonsense mutations that led to low low-density lipoprotein (LDL) levels and protection from coronary heart disease (Cohen et al., 2005, Cohen et al., 2006). Drugs now developed to inhibit *PCSK9* have a significant effect on multiple endpoints (Sabatine et al., 2017). The impact of *PCSK9* on drug development has been considerable, with human genetics now serving as a launching point for target discovery efforts in this field. More recently, Artificial Intelligence-based (AI) integration strategies that bring together genetic and other data modalities are pointing toward additional treatments for other conditions, such as TNIK inhibition for interstitial lung disease (Ren et al., 2025).

A similar story plays out in bone biology. WNT signaling was reported to affect skeletal patterning and development in 1994 (Wurst et al., 1994), but the coincident discoveries in 2002 that a 52 kb deletion downstream from *SOST* (an inhibitor of WNT signaling) impacting its expression was associated with van Buchem disease (Balemans et al., 2002) and that activating mutations in *LRP5* (a receptor for WNT) were causative for the autosomal high bone mass (Little et al., 2002) led to multiple attempts to develop therapies for bone disease that affected WNT signaling (Rauner et al., 2021; Jiang et al., 2022). Unlike for *PCSK9*, most of the drugs designed to modulate WNT signaling in the context of bone disease have either not made it to clinical trials, failed in clinical trials due to off-target effects, or remain yet unapproved for use (Rauner et al., 2021). The exception to this is Romosozumab. However, labeling by the FDA warns of increased risk for myocardial infarction, stroke, and cardiovascular death (Drug Approval Package: Evenity, n.d.). If human genetic evidence is key to finding efficacious targets and with examples like *PCSK9* or WNT signaling, bone genomics should be ripe with opportunity. Many bone-related phenotypes such as bone mineral density (BMD) (Alonso and Ralston, 2014), bone architecture (Karasik et al., 2017), and fracture incidence have high heritability (Karasik et al., 2017; Michaëlsson et al., 2005; Nethander et al., 2023), and therapies that improve BMD and prevent fracture should be amenable to genetic strategies. Numerous genetic associations have been discovered for BMD (Zhu et al., 2021). So why has the path been more challenging than with *PCSK9*?

## 2 The complexity of bone physiology and bone genetics

Bone is a complex metabolic tissue that is in a constant state of turnover. The osteoblast, a mesenchymal-derived cell type, makes and subsequently mineralizes the proteinaceous matrix of bone. The osteoclast, a myeloid-derived cell type, resorbs bone, including the matrix and the mineral component. The third major cell type of bone is the osteocyte. These complex cells are entombed in the bone and are derived from mature osteoblasts (Mohamed, 2008). Osteocytes appear to have bone forming and resorbing functions, in addition to their roles in regulating bone turnover and secreting hormones that control systemic phosphate metabolism (Tsourdi et al., 2018). Any chronic imbalance between bone formation and resorption in adulthood is generally associated with disease (Raisz, 2005). This complexity in bone physiology provides multiple points at which genetic factors can act and an equally dizzying number of points at which therapies may impact bone turnover.

The 2023 Nosology of Genetic Skeletal Disorders lists 771 inherited conditions that impact the skeleton, which are associated with 552 diseases (Unger et al., 2023). These genetic conditions are generally inherited in a classical Mendelian fashion, with one disease associated with one gene. The complexity arises because one gene may be associated with multiple disease presentations, depending on the nature of the variant. For example, variants in *FGFR3* are associated with five different chondroplasias, one bone overgrowth disorder, two forms of craniosynostosis, and one other patterning defect disorder (Unger et al., 2023). The diverse clinical presentation of genetic disorders of the skeleton often complicates genetic diagnosis, and other background genetic events that impact the primary disease-causing modification complicate the interpretation of genome sequencing results relative to clinical presentation (McCabe, 2017). Such modifier genes are understudied in skeletal biology.

Osteoporosis, a complex genetic disease, has long been appreciated to run in families. The phenotype of BMD, as determined by Dual X-ray Absorptiometry (DXA) (Kanis et al., 2008) or as estimated using other means such as quantitative ultrasound (Marín et al., 2006), has been the most widely used to understand the contribution of genetics to osteoporosis, as the heritability for peak BMD is estimated between 55 and 85% (Alonso and Ralston, 2014). Since the first Genome-wide association study (GWAS) for BMD in 2007, over 50 GWASs have been published for osteoporosis-related phenotypes (Zhu et al., 2021). To date, the largest single population BMD GWAS used data from the UK Biobank and identified 1103 independent genetic loci for the phenotype of estimated BMD. However, each locus only contributes a tiny amount to the overall phenotypic variance, and these loci accounted for ∼20 % of the heritability (Morris et al., 2019). GWASs using DXA-measured BMD have not enjoyed the large sample sizes available for estimated BMD. Indeed, in the UK Biobank, DXA BMD has only been measured on ∼10 % of participants. Using machine learning to infer missing DXA BMD data in the UK Biobank, Miao et al. identified an additional 89 loci but could not account for the full estimated heritability for BMD (Miao et al., 2024). Gene by Environment (G*E) interactions account for some missing heritability for other complex traits such as body mass index and plasma lipids (Boye et al., 2024; Herrera-Luis et al., 2024). G*E interactions in the field of bone biology are not unknown (Ackert-Bicknell et al., 2008; Kiel et al., 2007), but are another source of complexity that is understudied when considering the etiology of complex musculoskeletal diseases such as osteoporosis.

Many limitations remain from bone-related GWASs. First, while fractures are the clinical target, genetic studies of fracture incidence have identified fewer replicable associations, likely due to the additional influence of falls (Koromani et al., 2019). Second, BMD does not equate to “bone strength” as it does not provide information on bone “quality,” including bone material properties. Efforts to build genetic predictors of fracture were improved when multiple phenotypes derived from an electronic health record were integrated (Xiao and Wu, 2024), suggesting the potential of composite strategies to estimate bone strength. Third, while BMD captures bone size information, it does not fully capture bone shape information (Ott, 2016). There have been some attempts to capture non-BMD phenotypes such as shape in the GWAS (Faber et al., 2024; Karasik et al., 2012; Styrkarsdottir et al., 2019; Livingstone et al., 2021). The lack of readily available data sets in the public domain and the labor/time required to extract such information from DXA data, plus the limited availability of more sophisticated instruments and data collected in large cohorts using such instruments), such as HR-pQCT, which can measure true bone architecture in humans, have limited these efforts.

## 3 Artificial intelligence and machine learning terminologies and strategies

Artificial intelligence and machine learning (AI/ML) methods are computational strategies that are being deployed in biomedicine to integrate large data collections (Embedding and in biology., 2024). While AI is a broader class of algorithms than ML, many such methods come from a class of ML methods termed “deep learning” algorithms for their ability to combine input data into sets of composite features and then leverage those features to succeed at some desired tasks (Ching et al., 2018). These complex composite features integrate multiple elements from raw input data and go by different names depending on the underlying algorithm. Certain AI models are generative and can produce new hypothetical data that is distinct from but consistent with previous observations. Knowledge graphs, which integrate large knowledgebases into networks that can contain multiple types of entities and the relations between them, are an emerging tool to make sense of very complex observations (Hogan et al., 2021).

## 4 Potential applications of artificial intelligence and machine learning

### 4.1 Population diversity and representation in bone genomics

There is a phenomenon common in population genetics, computational biology, and AI techniques which is commonly referred to as the “streetlight effect”: evidence and insights are limited only to where a researcher looks. This is particularly apparent for *PCSK9*, as the loss-of-function variant is at appreciable frequencies in populations of African ancestry only: it would be difficult to identify the effect of this locus even in studies of hundreds of thousands to millions of non-African ancestry individuals (Ding and Kullo, 2008). This has the potential to limit and bias our insights in the field and contribute to the exacerbation of existing disparities (Martin et al., 2019). In particular, BMD and other bone phenotypes have been challenging to interpret clinically in some racial groups, and certain racial minorities are poorly represented or underrepresented in genetic and omics data sets (Cauley, 2011). As has been shown across the span of genome-wide association studies, the field suffers from a strong bias toward populations only of European ancestry (Bustamante et al., 2011; Popejoy and Fullerton, 2016). Indeed, most GWASs for bone have been conducted in a limited set of populations, and we have minimal information about the genetics of bone in other genetic similarity groups (Yau et al., 2021). This limits our discoveries to only the genetic variability that is present in that specific component of human genetic diversity. Critically, this has particular impacts on the study of rare genetic disorders, as the further down the allele frequency spectrum the more likely it is that variants remain population-private or restricted in their geographic distribution (Nelson et al., 2012; Keinan and Clark, 2012; Gravel et al., 2011). This problem is particularly apparent in human genetics due to the need to address population structure in standard analyses, and this is a significant problem across many modalities of biomedical research, leading to the U.S. Food and Drug Administration establishing a mandate of Diversity Action Plans for current and future clinical trials (Kozlov, 2023). To a certain extent, AI methods may be able to extrapolate across population structure but it's critical to consider AI fairness (avoidance of biases based on classifiers such as race or sex) (Zou and Schiebinger, 2018). An application of fair AI in this space could include developing algorithms for which individual-level predictions are appropriate for each person and not biased by demographics or other factors. A recent example of a biomedical algorithm in the musculoskeletal space designed to be fair showed that radiographic inference of knee osteoarthritis could have disparities reduced through the training of an appropriate algorithm (Pierson et al., 2021).

### 4.2 Drugs for metabolic bone disease, their limitations and new directions

Drug therapies currently available for treating metabolic bone disease either preserve the amount of bone present (primarily by suppressing bone resorption by the osteoclast) or stimulate the building of new bone by the osteoblast (Khosla and Hofbauer, 2017). There are currently eight categories of drugs approved for treating bone disorders, in Phase III clinical trials, or positioned to start clinical trial testing. Interestingly, at least one BMD GWAS identified the genes coding for the target(s) of all but one of these drugs (Zhu et al., 2021).

These drugs all target only a handful of molecular pathways, and each has advantages and, in some cases, severe limitations. The most prescribed antiresorptive drugs are the nitrogen-containing bisphosphonates that suppress bone resorption by disrupting the Farnesyl Phosphate pathway, thereby killing osteoclasts (Giusti and Papapoulos, 2018). Bisphosphonates are the only class of drugs not implicated by a GWAS to BMD (Zhu et al., 2021). Other antiresorptive drugs include denosumab (a monoclonal antibody that disrupts RANK/RANKL signaling), hormone replacement therapies (HRT) for postmenopausal women, and a set of selective estrogen receptor modulators (Zhu et al., 2021; Khosla and Hofbauer, 2017). A final set of antiresorptive drugs inhibit cathepsin K, but the only such drug to reach clinical trials showed promising effects on bone retention but failed in Phase III due to increased incidence of cardio-cerebrovascular events (Dai et al., 2020). Anabolic drugs currently approved for treating osteoporosis include teriparatide, abaloparatide, and romosuzumab. Teriparatide and abaloparatide work through the parathyroid hormone receptor and have strong fracture-prevention properties, but both are costly and have many contraindications (Zhu et al., 2021; Khosla and Hofbauer, 2017). There is mounting evidence from GWAS suggesting that teriparatide efficacy may be modified by G*E interaction(s) (Alonso et al., 2023). Romosozumab inhibits sclerostin (SOST), which itself inhibits pro-bone formation WNT signaling (di Filippo and Rosen, 2024). A final drug inhibits DKK1, another WNT signaling inhibitor, but has not yet reached clinical trials (Zhu et al., 2021). As a result, there remain substantial deficits in our ability to treat osteoporosis patients and those with other metabolic bone disorders, including some Mendelian skeletal diseases.

There are key limitations for all approved osteoporosis medications, including slight increases in risk for osteonecrosis of the jaw (ONJ) and subtrochanteric fractures in patients taking antiresorptives and a higher than expected incidence of patients discontinuing anabolic therapies due to adverse events (Ayers et al., 2023). While there are newer drugs becoming available for specific rare diseases in children, such as hypophosphatemic rickets (Goyal and Tandon, 2024), achondroplasia (Savarirayan et al., 2019), and severe forms of hypophosphatasia (Schindeler et al., 2024), the major drug class approved for use in children with bone fragility conditions remains bisphosphonates (Cheung, 2015). The use of bisphosphonates in children comes with the same concerns as for adults for ONJ but also includes additional complications in growing children, such as the formation of sclerotic bands at the growth plates and the development of persistent remodeling defects (Boyce et al., 2014). No anabolic drugs are currently approved for use in children.

Human genetics provides a potential path to develop new, safer, and more effective drugs for bone disease, especially for children. If *PCSK9* served as the Helen of Troy for human genetics in drug development - launching a thousand target discovery efforts - can artificial intelligence (AI) serve as Athena and guide these efforts home, at least in bone genomics? There are reasons to be hopeful. AI-based efforts in biology and medicine, spurred by interest in deep learning, have rapidly grown and have impacted many relevant areas, from structural modeling to variant interpretation (Ching et al., 2018). Throughout we provide examples of AI strategies that can aid in -omics-based target discovery. We connect these to key opportunities in bone genomics. We summarize how researchers could deploy such strategies to integrate complex data to reveal new targets with multifactorial human genetic support.

### 4.3 Identifying and connecting modules and pathways

In the case of *PCSK9*, variants in a single gene directly impacted a desired phenotype and, crucially, a loss of function in that gene produced a clinically beneficial effect. Traditional approaches, such as gene set enrichment analysis, are designed to connect pathways with experimental results (Subramanian et al., 2005), but they rely on curated gene sets that might constrain what can be discovered as relevant information remains unknown or not included in the database. AI/ML methods, which learn composite features, termed latent variables, which are complex variables created from patterns found across multiple observed data types may be helpful here. One such method, Pathway Level Information Extractor (PLIER) can align latent variables with curated gene sets or simply be observed in the training dataset (Mao et al., 2019). Subsequently, these methods have been applied to large collections of gene expression data to produce models with hundreds to thousands of signatures or previously unappreciated associations in the data (Taroni et al., 2019; Qiu et al., 2024).

**Fig. 1:**
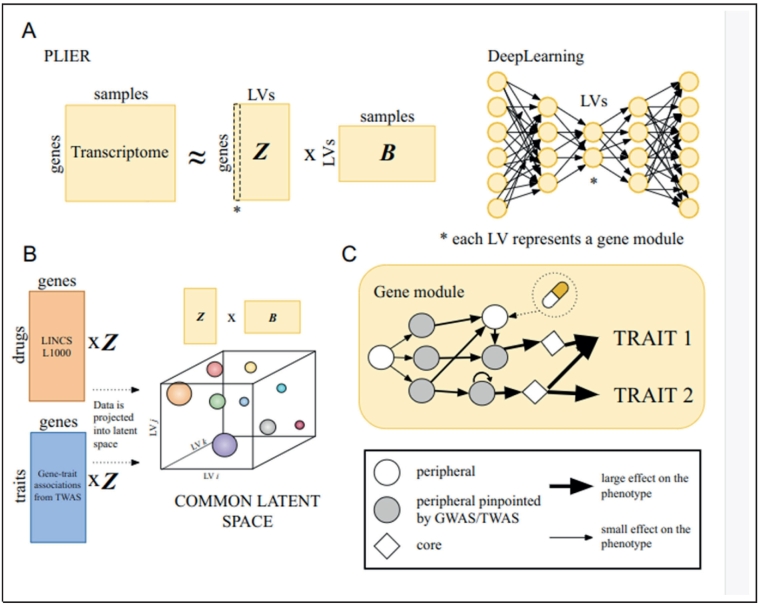
Identifying and connecting modules and pathways via machine-learning approaches. A. Two main strategies for extracting latent variables (LVs) from transcriptomic data. On the left, PLIER decomposes a gene-by-sample expression matrix into two matrices: Z (genes to latent variables) and B (latent variables to samples), with LVs representing co-expressed gene modules that can align with known pathways. On the right, deep learning (e.g., autoencoders) also infers LVs but can capture more complex, nonlinear relationships among genes. B. Once gene modules (LVs) are extracted, additional datasets—such as drug-induced transcriptional profiles (e.g., from LINCS L1000) or gene–trait associations (from TWAS)—are projected into the same latent space. This process creates a unified “common latent space,” linking phenotypes to putative pathways and drug mechanisms. C. In the omnigenic view, each gene module includes a mix of core genes (diamonds) that directly affect the phenotype and peripheral genes (circles) that indirectly modulate core genes through regulatory interactions. Arrows illustrate how peripheral genes influence core genes, ultimately altering traits. GWAS/TWAS may highlight some peripheral genes (gray circles), while others (white circles) remain undetected by single-gene approaches yet emerge through module-based analyses. Notably, a drug targeting a peripheral gene can still induce trait changes by triggering a regulatory cascade that propagates to core genes and, ultimately, to the phenotype.

These latent signatures facilitate mapping phenotypes to gene modules aligned with pathways and transcriptional processes, thereby extending our view of how complex traits emerge from interconnected regulatory networks. For instance, to study lipid accumulation in the liver, Pividori et al. projected gene-phenotype associations from transcriptome-wide association studies (TWAS) onto these latent variables and uncovered phenotype-relevant tissues (Pividori et al., 2023). Furthermore, by connecting phenotypes with these groups of functionally related genes, these methods can highlight therapeutic targets that do not emerge directly from GWAS or TWAS alone. For example, the projection strategy of Pividori et al. linked relevant traits with latent variables related to lipid phenotypes that not only captured TWAS-implicated genes but also included genes identified through a CRISPR screen for their direct effect on hepatocyte lipid accumulation (Pividori et al., 2023). Although these key genes, representing potentially more attractive targets, were not pinpointed by GWAS/TWAS, but were consistent with the omnigenic model that has been very useful for interpreting disease processes. The omnigenic model postulates that nearly all genes expressed in a disease-relevant cell can shape traits by modulating *core* genes (central control genes) through regulatory networks (Boyle et al., 2017). In this omnigenic view, *peripheral* genes (genes affected by the core gene) indirectly affect a phenotype via their influence on one or more *core* genes (Fig. 1). Such findings illustrate how AI/ML approaches expand the range of potential therapeutic targets, including more draggable genes or those more directly affecting a phenotype, by revealing the extensive network of genes that underlie complex phenotypes such as musculoskeletal diseases, including osteoporosis (Subirana-Granés et al., 2024).

In concept, finding these underappreciated targets is quite simple. The key to this approach was making an inferential link(s) in the space(s) between the observed biological data. The data measuring gene expression levels across many conditions was first compressed, using PLIER, into latent variables (Taroni et al., 2019). This produces complex composite features that remain connected to the underlying biological space observations. Second, data on variant-association relationships (biological space) was connected with variant-gene expression relationships (biological space) through an inferential step to produce variant-gene expression relationships (inferred space or inferred links) (Pividori et al., 2020). Finally, projecting these inferred relationships into the complex composite features learned by PLIER is an additional level of inference connecting latent variables with genetic associations that was key to identifying potential targets missed by association studies alone.

### 4.4 Expanding genetic evidence with cell-type insights

Evidence can also implicate a specific cell type in the processes underlying complex phenotypes, particularly musculoskeletal ones (Dillard et al., 2023). Single-cell RNA sequencing can reveal differences in cell type proportions or expression differences within cell types. Deconvolution methods use single-cell reference profiles with bulk profiling data and ML/AI techniques, often at a larger scale and from a targeted population, to estimate differences in cell type composition (Newman et al., 2019; Chu et al., 2022; Hu and Chikina, 2024). In other words, these methods use the information learned from single-cell data to infer what cells are represented in a bulk RNA seq sample. These methods will likely be most useful in settings where single-cell profiling is challenging - either in the context of rare samples (i.e., for rare diseases (Banerjee et al., 2023)) or where technical factors pose challenges (Hippen et al., 2023). This impact is particularly salient in single-cell studies of bone as current techniques rarely capture osteoclasts due to their size and the difficulties associated with dissociating mature osteoclasts from the bone surface. As a result, this cell class is often indirectly studied in single-cell studies by capturing their precursors in the marrow (Tsukasaki and Takayanagi, 2022). A similar issue affects the direct capture of marrow adipocytes, and marrow adipogenic lineage precursors (MALPs) often serve as a surrogate (Zhong et al., 2020). In other settings, researchers are beginning to identify and develop solutions to this missing cell type challenge (Ivich et al., 2024). Spatial transcriptomics has been modified now to allow for the collection of samples from calcified bone and has the potential to address some missing cell type issues, but as yet, spatial transcriptomics is not widely used and does yet suffer from limitations arising from resolution (Wehrle et al., 2024).

New methods seek to integrate bulk and single-cell data, not just to estimate cell composition, but to estimate differences in cell type gene expression, even when direct profiling of single cells is infeasible (Davidson et al., 2025). For example, Bulk Deconvolution with Domain Invariance (BuDDI) implements a strategy with generative AI to estimate cell type expression profiles in the context of a perturbation or sex difference. This method makes an inferrential leap by connecting observations of gene expression in single-cell data that lack the contrast of interest (biological space) with bulk RNA-seq data that contain the contrast of interest (biological space) to produce predicted cell-type specific expression profiles of the contrast of interest (inferred space) (Davidson et al., 2025). As noted above, there are limitations to the collection of replete samples for all cell types in bone for single-cell collection, and this method may prove valuable in this space as a result. BuDDI has not yet been applied to challenges of bone genomics, but application in the context of rare autoimmune disease implicated potential pathways and processes. A key benefit of integrating data using generative neural networks for hypothesis generation is that targeted experimental follow-up can be pursued, even when the large-scale profiling that would often guide such experiments is infeasible. As evidence of single-cell profiling's value for drug development accrues, such methods can help close the gap in difficult-to-assay settings (Dann et al., 2024).

### 4.5 Knowledge graph based explanation techniques

A key challenge in linking high-throughput findings to viable targets is understanding the strength of molecular evidence that underlies target-phenotype connections and, in particular, whether or not that evidence indicates an opportunity to intervene by targeting/interfering with a key node (i.e. a single metabolite in a complex metabolic pathway, or gene in a signaling pathway) in a network. Knowledge graphs provide an opportunity to evaluate the strength of evidence underlying potential target-phenotype paths rapidly (Nicholson and Greene, 2020). Several strategies inspired by AI/ML methods used for natural language processing of text have been developed to predict new edges (links between nodes) and classify paths in such networks. The node2vec strategy is one such option (Grover and Leskovec, 2016); many such strategies have been recently reviewed (Nicholson and Greene, 2020).

For explanation, hetnet connectivity search (Himmelstein et al., 2023) was developed to evaluate the strength of the evidence supporting connections between any two nodes in biomedical knowledge graphs. This method evaluates whether the number of paths identified is higher than expected in a null model that considers the degrees of the source and target nodes as well as the intermediate edge types. Such correction is important for biomedical applications - earlier work identified differences in degree distributions of systematically generated networks from those produced by curation, suggesting that differences due to degree might not be the most biologically informative (Zietz et al., 2024).

### 4.6 Maintaining rigor in AI/ML for bone genomics

A key challenge in applying AI/ML techniques is that incorrect application can lead to apparent strong performance while producing a low-quality set of predictions. In other words, the model performs very well, but the results are of little use. Whalen et al. provide critical guidance on using ML methods for genomics (Whalen et al., 2022). Key challenges often surface due to critical but unconsidered patterns in data - for example, whether or not technical sources of variation are confounded with an endpoint being predicted. In this context, it becomes more important that investigators share as much information as possible - including data, source code, and machine learning models - to maintain rigorous research practices (Heil et al., 2021). This need is balanced by the importance of maintaining participant privacy in certain genetic studies (Byrd et al., 2020). Taken together, investigators should share as much as possible publicly without compromising participant privacy, pairing that with controlled-access sharing where necessary. Maintaining these standards will allow bone genomics researchers to responsibly benefit from the opportunities that AI/ML techniques present.

## 5 Conclusion

In the long term, AI/ML strategies may usher in an era of precision bone medicine, potentially processing electronic health records (EHR), genetic, lifestyle, and wearable data to identify people who need treatment as early as possible, selecting the most appropriate therapy immediately, and monitoring the progress of interventions while changing strategies as needed. Between now and this learning-healthcare future, we expect a key impact of AI/ML in bone genomics to be refining the universe of potential target and therapeutic strategies. While *PCSK9* has demonstrated the potential promise for genetics in drug discovery and bone genomics is awash in potential findings, the bone genomics landscape is also incredibly complex. AI/ML methods can help sift through possibilities, identifying key convergence points where interventions may be particularly effective.

## CRediT authorship contribution statement

**Casey S. Greene:** Writing – review & editing, Writing – original draft, Conceptualization. **Christopher R. Gignoux:** Writing – review & editing. **Marc Subirana-Granés:** Writing – review & editing, Visualization. **Milton Pividori:** Writing – review & editing. **Stephanie C. Hicks:** Writing – review & editing. **Cheryl L. Ackert-Bicknell:** Writing – review & editing, Writing – original draft, Conceptualization.

## Declaration of Generative AI and AI-assisted technologies in the writing process

During the preparation of this work the author(s) used ChatGPT to suggest structural improvements and improve readability for a broad audience. After using this tool/service, the authors reviewed and edited the content as needed and take full responsibility for the content of the publication.

## Declaration of competing interest

The authors declare the following financial interests/personal relationships which may be considered as potential competing interests: CSG reports financial support was provided by the National Institutes of Health (R01 HG010067 and R01 HD109765). CRG reports financial support was provided by the National Institutes of Health (R01 HG011345). MP reports financial support was provided by the National Institutes of Health (R00 HG011898 and R01 HD109765). CLAB reports that financial support was provided by the National Institutes of Health (R01 AR082880 and AR079839).CLAB reports a relationship with BioMarin Pharmaceutical Inc. unrelated to this work that includes: funding grants. CLAB served as a guest editor for this issue of Bone Reports. If there are other authors, they declare that they have no known competing financial interests or personal relationships that could have appeared to influence the work reported in this paper.

## Data Availability

No data was used for the research described in the article.
